# Modeling and Experimental Validation of a Bionic Underwater Robot with Undulating and Flapping Composite Propulsion

**DOI:** 10.3390/biomimetics10100678

**Published:** 2025-10-09

**Authors:** Haisen Zeng, Minghai Xia, Qian Yin, Ganzhou Yao, Zhongyue Lu, Zirong Luo

**Affiliations:** 1 College of Intelligence Science and Technology, National University of Defense Technology, Changsha 410073, China; 2 National Key Laboratory of Equipment State Sensing and Smart Support, National University of Defense Technology, Changsha 410073, China; 3 China Aerodynamics Research and Development Center, Mianyang 621000, China; 4 College of Energy and Power Engineering, Changsha University of Science and Technology, Changsha 410076, China

**Keywords:** bionic underwater robot, compound drive, undulating fin, flapping wing, computational fluid dynamics, hydrodynamic simulation

## Abstract

As the demand for marine resource development escalates, underwater robots have gained prominence as a technological alternative to human participation in deep-sea exploration, resource assessments, and other intricate tasks, underscoring their academic and engineering importance. Traditional underwater robots, however, typically exhibit limited resilience to environmental disturbances and are readily obstructed or interfered with by aquatic vegetation, sediments, and other physical impediments. This paper examines the biological locomotion mechanisms of black ghostfish, which utilize undulatory fins and flapping wings, and presents a coupled undulatory-flapping propulsion strategy to facilitate rapid movement and precise posture adjustment in underwater robots. A multimodal undulatory-flapping bio-inspired underwater robotic platform is proposed, with a systematic explanation of its structure and motion principles. Additionally, kinematic and dynamic models for coordinated propulsion with multiple actuators are developed, and the robot’s performance under various driving modes is evaluated using computational fluid dynamics simulations. The simulation outcomes confirm the viability of the developed dynamic model. A prototype was constructed, and a PID-based control algorithm was developed to assess the robot’s performance in linear movement, turning, and other behaviors in both undulatory fin and flapping modes. Experimental findings indicate that the robot, functioning in undulatory fin propulsion mode at a frequency of 2.5 Hz, attains a velocity of 0.35 m/s, while maintaining attitude angle fluctuation errors within ±5°. In the flapping propulsion mode, precise posture modifications can be executed. These results validate the feasibility of the proposed multimodal bio-inspired underwater robot design and provide a new approach for the development of high-performance, autonomous bio-inspired underwater robots.

## 1. Introduction

The Earth’s surface is more than 70% covered by oceans, which are home to a substantial amount of strategic resources, including biological, mineral, and oil and gas resources [1,2]. Nevertheless, the marine environment is distinguished by extreme temperatures, low visibility, high hydrostatic pressure, and strong currents, all of which present substantial obstacles to resource exploration and development [3]. Underwater robots have emerged as a critical technological tool for the replacement of humans in the performance of environmental monitoring, resource surveying, and deep-sea exploration. This technology has the potential to significantly enhance research value and practical applications. Tasks such as emergency rescue, marine environmental monitoring, underwater engineering construction, and seabed resource exploration can be performed by underwater robots in the civilian sector. In the military sector, they can perform high-value duties such as reconnaissance surveillance, anti-submarine warfare, mine clearance, and underwater communication support [4,5]. Therefore, the development of high-performance, multifunctional bio-inspired underwater robots has become an essential research direction in the field of marine engineering technology. Traditional propeller-driven underwater robots face several limitations in complex environments, such as susceptibility to entanglement, high noise levels, intense water disturbances, and limited endurance. In contrast, bio-inspired underwater robotics offer significant advantages in terms of stealth, environmental adaptability, and propulsion efficiency [6]. However, single-mode bio-inspired propulsion systems still have certain limitations, such as insufficient maneuverability of undulatory fin propulsion in complex terrain and low efficiency of flapping wing propulsion at high velocities [7,8]. Therefore, multimodal composite drive modes that integrate multiple motion mechanisms demonstrate significant advantages in terms of high maneuverability, strong environmental adaptability, and energy efficiency, holding great theoretical significance and practical value.

Scholars worldwide have conducted extensive research on undulatory fin propulsion. The Low team at Nanyang Technological University developed the Nanyang Knifefish (NKF-II) modular robot [9]. The robot employs the knifefish as a biomimetic template to achieve arbitrary waveform control by connecting fin rays through serial sliders. In the same vein, the knifefish was utilized as a biomimetic template by the team of Hanlin Liu and Oscar Curet at Florida Atlantic University to develop KnifeBot, an underwater automaton [10]. Sixteen servomotors regulate the fin undulations, which allows the robot to execute complex maneuvers such as vertical swimming, forward and rearward movement, and the preservation of a fixed position. Bao Haime’s team at Dalian Minzu University [11] created a two-degree-of-freedom undulatory fin propulsor that was influenced by the biomimetic characteristics of the electric eel. The propulsor is designed with flexible silicone coatings and carbon fiber materials, which ensure both durability and lightweight properties. The design of an actively controlled bio-inspired pectoral fin, which was propelled by a single motor, significantly reduced the complexity and weight of the drive system in the study of bi-directional undulatory fin bio-inspired underwater robots by teams from Zhejiang University and other institutions [12]. The cuttlefish served as the biomimetic paradigm for the dual-side undulatory fin robot, which was developed by Li Yuhao’s team at Chongqing University [13]. The flexible undulatory fins, which are powered by multiple actuators, enable the robot to achieve exceptional maneuverability. Wang He’s team at the National University of Defense Technology [14] created a tri-undulatory amphibious bio-inspired robot by combining the locomotion capabilities of rays and snakes. The robot replicates the undulatory propulsion of stingrays by utilizing flexible fan-shaped appendages and the serpentine motion of snakes to enable efficient locomotion in both aquatic and terrestrial environments.

Maria L. Castaño’s team at Johns Hopkins University [15] developed a bio-inspired underwater robot that is propelled by a paddle-style pectoral fin in their research on fluttering propulsion mode. This robot generates oscillation around the vertical axis by employing a rigid pectoral fin and a single-degree-of-freedom rotational joint. Taesik Kim and associates at Pohang University of Science and Technology [16] introduced the “HERO-BLUE” robot, which incorporates soft undulatory fins, a flexible spine inspired by salamanders, and multimodal fins. This design enables the robot to move in a multimodal manner, including swimming, walking, and crawling. The U-CAT underwater robot, which was created by Ahmed Chemori’s team at Tallinn University of Technology [17], is capable of achieving high maneuverability and minimal disturbance during motion by utilizing four oscillating fins for bio-inspired propulsion. The PEAR amphibious underwater robot, which was developed by Son-Cheol Yu’s team at Pohang University of Science and Technology, Korea [18], employs a hinged multimodal paddle system to perform both underwater walking and swimming. 

To comprehensively assess the potential applications of different propulsion systems in underwater vehicles, this study conducts a comparative analysis of three mainstream methods: propeller propulsion, oscillating propulsion, and flapping propulsion. The evaluation criteria include propulsion efficiency, maximum navigation speed, environmental adaptability, maneuverability, and structural complexity, employing a systematic quantitative approach for assessment. A scoring system is utilized, with all five criteria rated on a scale from 1 to 5. For propulsion efficiency and maximum speed, a score of 1 indicates the lowest performance, while a score of 5 indicates the highest. In terms of environmental adaptability and maneuverability, a score of 1 represents the weakest performance, whereas a score of 5 signifies the strongest. Regarding structural complexity, a score of 1 denotes the most complex design, while a score of 5 indicates the simplest.

**Figure 1:**
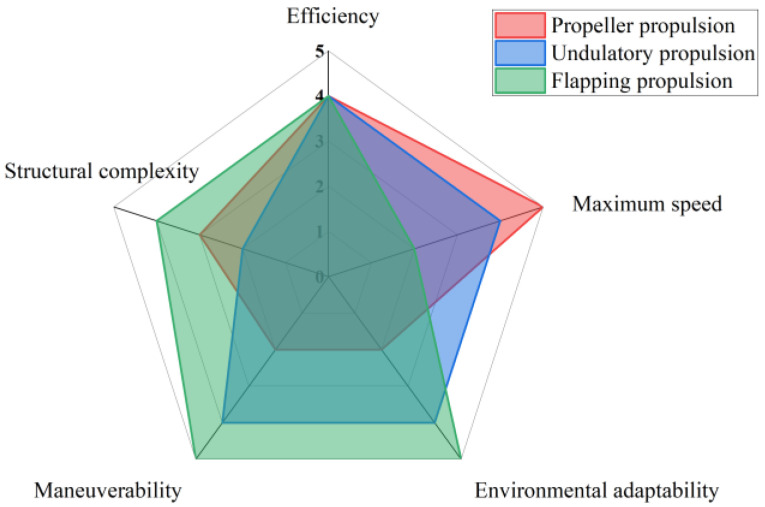
Comparative analysis of performance indicators under three driving modes.

As illustrated in Figure 1, the propeller propulsion system is a mature technology that offers significant advantages in high-speed cruising, characterized by relatively high propulsion efficiency, advanced technological maturity, and strong reliability. However, propeller systems are associated with high noise levels and are prone to entanglement with aquatic vegetation and debris, which adversely affects their adaptability to environmental conditions. Additionally, their maneuverability is constrained in complex environments due to inherent design limitations. The structure of propeller propulsion systems is relatively complex, necessitating regular maintenance and protective measures.

In contrast, oscillating propulsion systems, with their biomimetic design, demonstrate superior performance and high propulsion efficiency in low-speed and complex aquatic environments. These systems exhibit good adaptability to varying water conditions and possess excellent maneuverability, enabling flexible steering and attitude adjustments. Typically composed of multiple repetitive swinging components that generate complex waveforms, oscillating propulsion systems have a relatively intricate structure. While they can achieve lower energy consumption in low-speed and complex environments, energy consumption significantly increases during high-speed operation.

Flapping propulsion systems, as another form of biomimetic propulsion, exhibit outstanding performance in terms of environmental adaptability and maneuverability. Their high efficiency is derived from mimicking biological movements, allowing them to adapt effectively to complex environments. Flapping propulsion systems can respond flexibly in challenging conditions and possess excellent control and maneuverability, enabling precise operations. Despite their relatively simple structure and ease of integration, energy consumption tends to increase significantly during high-speed and high-intensity maneuvers.

In summary, different propulsion systems have their respective advantages and disadvantages, making them suitable for various applications. Propeller propulsion is ideal for long-distance high-speed travel in environments where adaptability is not a primary concern; oscillating propulsion is better suited for short-distance, low-speed navigation where noise reduction and maneuverability are critical; while flapping propulsion is most appropriate for complex aquatic environments requiring high adaptability and maneuverability. In practical applications, it is essential to consider specific task requirements and environmental conditions to select the most suitable propulsion system.

Despite substantial advancements in conventional propeller-based propulsion and biomimetic approaches such as undulatory and flapping fin propulsion [19,20], these methodologies predominantly employ single-modal drive systems [21]. Such systems are characterized by inherent limitations in maneuverability, imbalanced energy consumption, and insufficient adaptability to complex terrains. While research into multimodal propulsion has begun to emerge, there remains a conspicuous paucity of work that systematically integrates multiple modalities, such as undulatory and flapping fins, and analyzes them within a unified hydrodynamic framework. Consequently, a comprehensive solution capable of simultaneously achieving high maneuverability, robust environmental adaptability, and exceptional energy efficiency in complex underwater environments has yet to be fully realized.

In light of these research deficiencies, this paper proposes a novel multimodal composite drive paradigm for underwater robots, drawing inspiration from the biological prototype of the electric knifefish. This design synergistically combines undulatory fin propulsion with flapping fin propulsion, positioning the undulatory fin as the core driver for primary locomotion and the flapping fins as auxiliary actuators for enhanced maneuverability and posture control. This integrated approach aims to facilitate high maneuverability, strong environmental adaptability, and superior energy efficiency during locomotion in complex aquatic settings. The core innovations of this paper include the following:

(1) This study details the design and construction of a bimodal coupled composite-driven biomimetic underwater robot platform, inspired by the locomotion principles of the electric knifefish. The platform features a biomimetic undulatory fin situated on the ventral side of the body for efficient primary propulsion, complemented by two pairs of biomimetic flapping fins mounted laterally to achieve precise attitude adjustment and auxiliary thrust. Crucially, the system enables synergistic composite propulsion between the undulatory and flapping fins.
(2) To substantiate the design’s credibility, we have established comprehensive kinematic and dynamic models of the robot. Concurrently, we have devised corresponding motion control and power distribution strategies. Through theoretical analysis, we have systematically discussed the robot’s characteristics under both straight-line and turning maneuvers. Simulation results underscore the capability of the undulatory fin to generate controllable vectorial thrust.
(3) Experimental validation of a proof-of-concept prototype further corroborates the feasibility of this composite drive mode. In conjunction with diverse motion control strategies, the robot demonstrably exhibits agile switching between locomotion modes, including forward movement, turning, and depth adjustment, across a variety of scenarios. Notably, when the undulatory fin operates at a frequency of 2.5 Hz, a maximum velocity of 0.35 m/s is achieved, while the flapping fins effectively perform attitude regulation functions at low speeds.

The remainder of this paper is organized as follows: Section 2 elaborates on the proposed multimodal composite drive strategy, encompassing the design philosophy, key technologies, and specific structural implementations. Section 3 presents the theoretical modeling and simulation framework for the robot, covering kinematic and dynamic analyses, as well as simulation verification methodologies. Section 4 details the construction of the experimental platform and presents the simulation results alongside experimental design and motion performance test outcomes, followed by an analysis and discussion of critical experimental data and phenomena. Section 5 delves into a comprehensive discussion of the findings, outlining directions for future improvements and potential application scenarios. Finally, Section 6 provides a summary of the entire work, enumerating the main contributions and outlining future research trajectories.

## 2. Bio-Inspired Design and Locomotion Mode

### 2.1. Bio-Inspired Design

**Figure 2:**
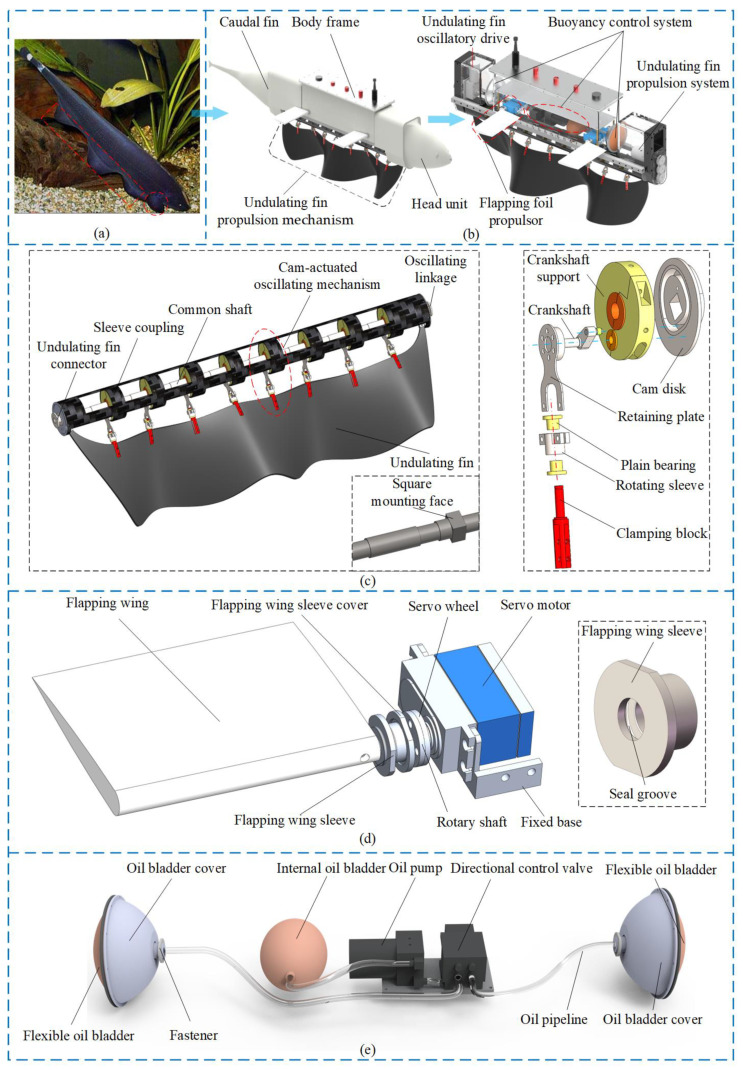
Principles and structural design of multimodal bio-inspired underwater robots. (**a**) Biomimetic model; (**b**) Biomimetic underwater robot propulsion platform; (**c**) Bio-inspired flexible undulatory fin propulsion device; (**d**) Bionic flapping wing mechanism with a single degree of freedom; (**e**) Bionic buoyancy control mechanism.

The primary design objectives of this paper are maneuverability, flexibility, and concealment, with the knifefish serving as the biomimetic model (as illustrated in Figure 2a). This paper introduces a bio-inspired underwater robot motion platform that is based on undulatory-flapping multimodal composite motion. The platform is designed to facilitate rapid forward, backward, and turning movements in confined spaces, and it utilizes flapping wings to fine-tune posture. Figure 2b illustrates this concept. Figure 2b’s left side illustrates the robot’s overall appearance, which is derived from the knifefish’s elongated, streamlined body. It comprises a head unit, body frame, caudal fin, undulating fin, and undulating fin propulsion device. The head and tail are predominantly designed to reduce drag and offer protection. The primary body of the robot, which comprises the undulating fin propulsion system, buoyancy control system, undulating fin oscillatory drive device, and flapping foil propulsor, is illustrated on the right side of Figure 2b. This robot is capable of gliding and possesses fundamental movement capabilities, including buoyancy control, guidance, and forward motion. It has the potential to be extensively implemented in the fields of subsea cable inspections, pipeline monitoring, human–machine collaborative operations, and underwater resource exploration, with the objective of overcoming the conventional technical constraints of limited maneuverability and short endurance in bio-inspired underwater robots.

To mimic the undulatory fin effect of the knifefish, a bio-inspired flexible undulatory fin propulsion device based on a cam mechanism was designed, consisting of an undulatory connecting rod, a common axis for the sleeve, a cam oscillation mechanism, oscillation connecting pieces, and an undulatory fin. The motor drives the undulatory connecting pieces to rotate, which in turn drives the common axis to rotate and further activates eight cam oscillation mechanisms with a 90° phase difference, achieving the undulatory movement of the fin (as shown in Figure 2c). In the cam oscillation mechanism, the cam disk is solidly connected to the common axis via a square mounting surface. The swing arm uses a crankshaft design, with one end embedded in the cam groove and the other end fixed to a fixed plate. The swing arm is hinged via a support bracket, converting the continuous rotational movement of the cam into periodic oscillations at the end of the swing arm. The fixed plate is solidly connected to the rotating sleeve of the adaptive rotating mechanism. Under the action of a sliding bearing, the clamping block can adapt to changes in the tangent direction of the undulatory fin, preventing rigid fin rays from cutting the waveform. One end of the undulatory connecting piece is fixed to the common axis, while the other end is connected to the coupling of the undulatory fin mechanism, realizing the undulation of the fin surface. One end of the oscillation connecting piece is solidly connected to the sleeve, while the other end is connected to the coupling of the undulatory fin oscillation device, achieving the overall oscillation of the fin body.

The line-fin electric eel features flexible undulating fins at its base and pectoral fins on either side of its body, which assist in maintaining balance and facilitating minor posture adjustments. A bionic flapping wing mechanism with a single degree of freedom was designed based on this principle. It consists of a servo motor, a rudder disk, a wing sleeve, a wing, a wing sleeve cap, a fixed base, and a rotating shaft, as illustrated in Figure 2d. The servo motor is connected to the body through a fixed base and, via the rudder disk and rotating shaft, facilitates the single-degree-of-freedom rotation of the flapping wing. The wing sleeve cover and the wing sleeve are affixed to the body, with both components featuring sealing grooves and equipped with sealing rings to maintain the seal between the body, rotating shaft, and sleeve. Considering robotic motion symmetry and control simplification, the four flapping wings are arranged symmetrically in the fore and aft directions. This configuration ensures robust performance across various motion states, including forward movement, backward movement, and turning.

A bionic buoyancy control mechanism has been developed, inspired by the principle of buoyancy regulation via volume adjustment in the fish bladder. This mechanism comprises an oil bladder cover, internal oil bladders, an oil pump, a valve, a deformable oil film, oil pipes, and fasteners, as illustrated in Figure 2e. The oil bladders located in the robot’s head, tail, and body facilitate the intake and discharge of hydraulic oil through the operation of the oil pump, thereby adjusting the robot’s buoyancy by altering its volume. The directional valve independently regulates the oil volume of the external oil bladders, thereby facilitating adjustments to the robot’s pitching posture.

**Figure 3:**
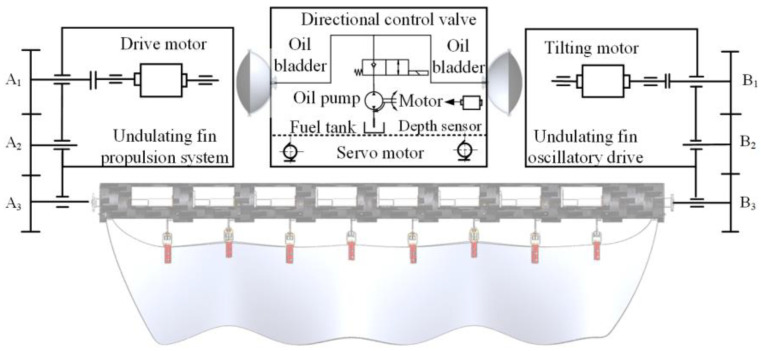
Schematic diagram of underwater robot drive principle.

Figure 3 illustrates the transmission mechanism principle for the multi-modal composite-driven underwater robot. The undulating fin oscillation mechanism operates through a high-torque servo motor and gear transmission system, facilitating the deflection of the fins for maneuvers like turning. The undulating fin oscillation system is powered by a high-speed, high-torque motor coupled with a gear transmission system, which drives the undulating fin shaft to produce the fin surface’s undulating movement. The buoyancy control system, governed by a depth sensor, employs a motor to operate the hydraulic pump and valve, thereby regulating the flow of hydraulic oil and facilitating the robot’s buoyancy control. The flapping wing mechanism on either side of the body employs servos to actuate the wings, facilitating a single-degree-of-freedom swinging motion.

### 2.2. Multi-Modal Motion of Composite-Driven Underwater Robots

This study proposes a composite drive architecture utilizing undulating fins and flapping wings to meet the multi-dimensional requirements of complex underwater tasks. The coordination of dynamic distribution among multiple actuators enables the intelligent switching between typical motion modes, including forward, backward, and yawing.

#### 2.2.1. Forward and Backward Motion

**Figure 4:**
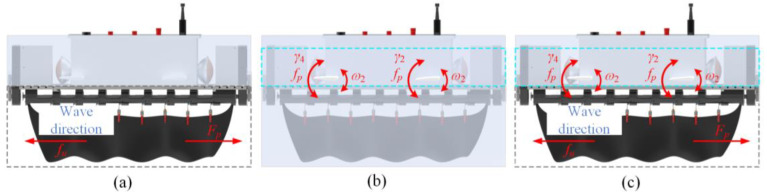
Forward and backward motion drive mode. (**a**) Undulating fin propulsion mode; (**b**) Flapping wing micromotion mode; (**c**) Composite drive mode.

The dynamic performance of forward and backward motion, as the primary mode of operation for underwater robots, directly affects the efficiency of task execution. This system offers three drive modes based on different operational conditions: undulating fin drive, flapping wing drive, and composite drive mode. Figure 4 illustrates the driving strategy schematics for each mode.

- Undulating Fin Propulsion Mode

Figure 4a illustrates the robot operating in the undulating fin propulsion mode, wherein movement is facilitated by the undulation of the fins, optimized for high-speed cruising in open waters. This mode produces thrust via the traveling wave motion of the undulating fins, facilitating the robot’s movement in both forward and backward directions. The strategy for power distribution is outlined as follows:

(1) $$\left\{\begin{array}{l} \gamma_{i}(t)=0,\forall i\in [1,4]\\ F_{P}(t)=\pm F_{P}\\ \rho (t)=0\\ f_{u}(t)\in [1,5]\;\mathrm{Hz} \end{array}\right.$$

In this context, $\gamma_{i}(t)$ denotes the flapping amplitude of the four flapping wings, $F_{P}(t)$ is the thrust generated by the undulating fins, ρ(t) is the overall deflection angle during the undulation phase, and $f_{u}(t)$ represents the oscillation frequency of the undulating fins.

- Flapping Wing Micromotion Mode

Figure 4b depicts the flapping wing drive mode, wherein the robot’s locomotion is facilitated by the periodic flapping of its wings, rendering it appropriate for near-field tasks such as biological observation. In this mode, the flapping wing mechanism generates periodic lateral jet thrust, which is related to the flapping amplitude γ(t), swing amplitude, and the flapping frequency *f_p_*. The power allocation strategy is as follows:

(2) $$\left\{\begin{array}{l} \gamma_{i}(t)=\pm \gamma_{0}\sin(2\pi f_{p}t),\forall i\in [1,4]\\ F_{P}(t)=0\\ \rho (t)=0\\ f_{p}\in [1,5]\;\mathrm{Hz} \end{array}\right.$$

- Composite Drive Mode

Figure 4c depicts the composite drive mode, which combines the movements of undulating fins and flapping wings. The undulating fins serve as the main source of propulsion, while the flapping wings aid in maintaining posture, rendering it suitable for executing precise tasks in dynamic environments characterized by disturbances like turbulence. This mode integrates the primary propulsion from the undulating fins with the secondary modulation offered by the flapping wings, resulting in enhanced motion efficiency. The power allocation strategy is outlined as follows:

(3) $$\left\{\begin{array}{l} \gamma_{i}(t)=\pm \gamma_{0}\sin(2\pi f_{p}t),\forall i\in [1,4]\\ F_{P}(t)=\pm F_{P}\\ \rho (t)=0\\ f_{u}(t)\in [1,5]\;\mathrm{Hz}\\ f_{p}\in [1,5]\;\mathrm{Hz} \end{array}\right.$$

#### 2.2.2. Steering Motion

Steering motion serves as the primary mechanism for heading adjustment in underwater robots, with its dynamic response characteristics significantly affecting maneuverability in complex flow environments. This study presents three yaw drive modes—undulating fin drive, flapping wing differential drive, and composite drive mode—to address the diverse requirements for yaw motion in underwater robots.

- Undulating Fin Propulsion Mode

**Figure 5:**
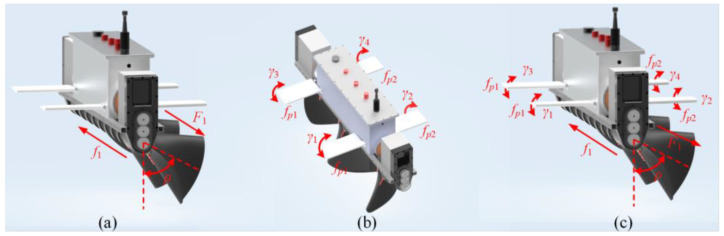
Steering motion drive mode. (**a**) Undulating fin propulsion mode; (**b**) Flapping wing micromotion mode; (**c**) Composite drive mode.

Figure 5a depicts the yaw motion associated with undulating fin drive. This mode provides significant dynamic torque output and robust resistance to flow disturbances, rendering it suitable for high-speed motion situations, including emergency obstacle avoidance and large-angle heading adjustments. In this mode, steering motion is achieved by generating an asymmetric flow field through the adjustment of the deflection angle ρ(t) of the undulating fins. The power allocation strategy is as follows:

(4) $$\left\{\begin{array}{l} \gamma_{i}(t)=0\\ F_{P}(t)=\pm F_{P}\\ \rho (t)\neq 0\\ f_{u}(t)\in [1,5]\;\mathrm{Hz} \end{array}\right.$$

- Flapping Wing Micromotion Mode

Figure 5b depicts the differential mode of the flapping wing. This mode employs differentiated motion of the left and right flapping wings to circumvent water disturbances associated with frequent adjustments of the deflection angle of undulating fins, rendering it appropriate for low fluid disturbance environments, such as biological tracking. This mode utilizes a differential control method, enabling the flapping wings on the robot’s left and right sides to operate at varying flapping angles or frequencies, thereby facilitating differential steering through the flapping wings. The power allocation strategy is outlined as follows:

(5) $$\left\{\begin{array}{l} \gamma_{1,3}(t)=\gamma_{0}\left(\sin(2\pi f_{p}t)\mp \Delta \gamma \right)\\ \gamma_{2,4}(t)=\gamma_{0}\left(\sin(2\pi f_{p}t)\pm \Delta \gamma \right)\\ F_{P}(t)=0\\ \rho (t)=0\\ f_{p}\in [1,5]\;\mathrm{Hz} \end{array}\right.\;\mathrm{or}\;\left\{\begin{array}{l} \gamma_{i}(t)=\pm \gamma_{0}\sin(2\pi f_{p}t),\;\forall i\in [1,4]\\ F_{P}(t)=0\\ \rho (t)=0\\ f_{p1}\neq f_{p2} \end{array}\right.$$

Here, Δγ represents the differential amount, and $f_{p1}$ and $f_{p2}$ are the flapping frequencies of the left and right flapping wings, respectively.

- Composite Drive Mode

Figure 5c depicts the composite drive mode, integrating the motion of undulating fins with flapping wings. The undulating fins serve as the primary means of propulsion, whereas the flapping wings facilitate posture adjustment, rendering it suitable for precise operations in dynamic environments characterized by substantial water flow disturbances. This mode combines the lateral thrust produced by the undulating fins with the differential steering of the flapping wings, resulting in a steering torque through their synergistic interaction. The strategy for power allocation is outlined as follows:

(6) $$\left\{\begin{array}{l} \gamma_{1,3}(t)=\gamma_{0}\left(\sin(2\pi f_{p}t)\mp \Delta \gamma \right)\\ \gamma_{2,4}(t)=\gamma_{0}\left(\sin(2\pi f_{p}t)\pm \Delta \gamma \right)\\ F_{P}(t)=\pm F_{P}\\ \rho (t)\neq 0\\ f_{u}(t)\in [1,5]\;\mathrm{Hz}\\ f_{p}\in [1,5]\;\mathrm{Hz} \end{array}\right.$$

## 3. Theory and FEM-Based Modeling

### 3.1. Theory Modeling

**Figure 6:**
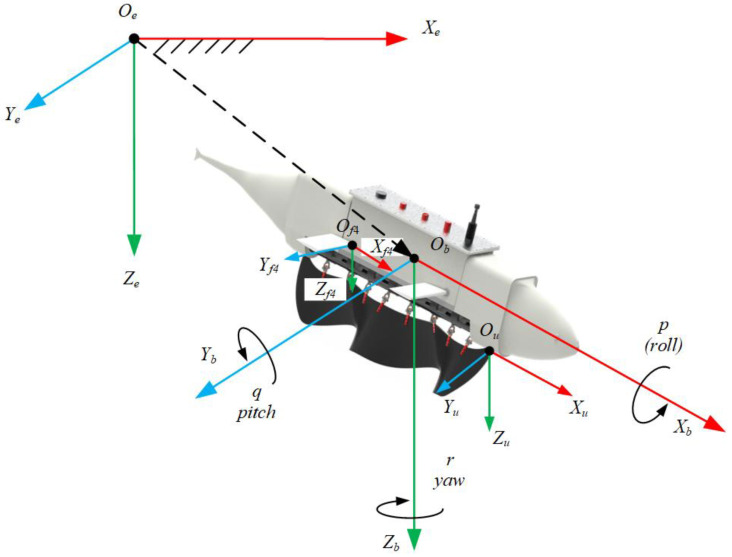
Bionic underwater robot kinematics.

To accurately describe the motion process of underwater robots in underwater environments, it is necessary to establish the robot’s kinematic model, undulating fin motion equations, and flapping wing motion equations, as shown in Figure 6. First, establish the global coordinate system $O_{e}-X_{e}Y_{e}Z_{e}$, where the coordinate point $O_{e}$ is any point inside the Earth, the $O_{e}Z_{e}$ axis points to the Earth’s center, and the $O_{e}X_{e}$ and $O_{e}Y_{e}$ axes follow the right-hand rule. Next, establish the robot coordinate system $O_{b}-X_{b}Y_{b}Z_{b}$, with the origin fixed at the robot’s center of mass. The $O_{b}X_{b}$ axis is the robot’s primary symmetry axis, the $O_{b}Y_{b}$ axis aligns with the secondary symmetry axis, and the $O_{b}Z_{b}$ axis is determined by the right-hand rule.

Define the undulating fin coordinate system $O_{u}-X_{u}Y_{u}Z_{u}$, with the origin at the base of the undulating fin, pointing forward. The $O_{u}-X_{u}$ axis points forward along the baseline of the undulating fin. The $O_{u}Y_{u}$ axis is perpendicular to the fan direction of the undulating fin, and $O_{u}Z_{u}$ is defined by the right-hand rule.

Finally, establish the flapping wing coordinate system $O_{f}-X_{f}Y_{f}Z_{f}$, with the origin $O_{f}$ located at the short apex of the flapping wing. The $O_{f}Z_{f}$ axis is perpendicular to the flapping wing plane, the $O_{f}Y_{f}$ axis is parallel to the long edge of the flapping wing, and the $O_{f}X_{f}$ axis is determined by the right-hand rule. Therefore, four flapping wing coordinate systems can be established, which are $O_{f1}-X_{f1}Y_{f1}Z_{f1}$, $O_{f2}-X_{f2}Y_{f2}Z_{f2}$,$O_{f3}-X_{f3}Y_{f3}Z_{f3}$ and $O_{f4}-X_{f4}Y_{f4}Z_{f4}$.

The pose information *η* of the underwater robot in the global coordinate system consists of the position vector *η*_1_ and the attitude vector *η*_2_, expressed as *η* = [*η*_1,_ *η*_2_]*^T^*. Here, *η*_1_ = [*X*,*Y*,*Z*]*^T^* represents the three-dimensional position of the vehicle in the inertial coordinate system, while *η*_2_ = [*φ*,*θ*,*ψ*]*^T^* denotes the attitude angles, describing the orientation of the vehicle relative to the inertial frame. The velocity vector *v* in the body coordinate system is composed of the linear velocity vector ($v_{1}^{T}$) and the angular velocity vector ($v_{2}^{T}$), i.e., *v* = [$v_{1}^{T}$,$v_{2}^{T}$]*^T^*; where *v*_1_ = [*u*,*v*,*w*]*^T^* represents the projections of the linear velocity vector along the *O_b_X_b_*, *O_b_Y_b_*, *O_b_Z_b_* axes of the robot’s coordinate system, *v*_2_ = [*p*,*q*,*r*]*^T^* represents the projection of the angular velocity vector on the *O_b_X_b_*, *O_b_Y_b_*, *O_b_Z_b_* axes of the robot’s coordinate system.

The kinematic model of the bionic underwater robot delineates the correlation between pose information and the velocity vector. Consequently, the kinematic equation of the robot within the inertial frame is as follows:

(7) $$\dot{\eta }=J(\eta )v$$

Here, *J*(*η*) represents the velocity transformation matrix from the body coordinate system to the inertial coordinate system, expressed using Euler angles.

For a rigid body in space, the velocity transformation matrix *J*(*η*) can be expressed as follows:

(8) $$J(\eta )=\left(\begin{array}{cc} J_{1}(\eta ) & 0\\ 0 & J_{2}(\eta ) \end{array}\right)$$

In this case, $J_{1}\left(\eta \right)$ and $J_{2}(\eta )$ represent the rotation matrices for linear and angular velocities, respectively. Their forms are as follows:

(9) $$J_{1}(\eta )=\left[\begin{array}{ccc} \cos\psi \cos\theta & \cos\psi \sin\theta \cos\phi -\sin\psi \cos\phi & \cos\psi \sin\theta \cos\phi +\sin\psi \sin\phi \\ \sin\psi \cos\theta & \sin\psi \sin\theta \cos\phi +\cos\psi \cos\phi & \sin\psi \sin\theta \cos\phi -\cos\psi \sin\phi \\ -\sin\theta & \cos\theta \sin\phi & \cos\theta \cos\phi \end{array}\right]$$

(10) $$J_{2}(\eta )=\left[\begin{array}{ccc} 1 & \tan\theta \sin\phi & \tan\theta \cos\phi \\ 0 & \cos\phi & -\sin\phi \\ 0 & \sec\theta \sin\phi & \sec\theta \cos\phi \end{array}\right]$$

#### 3.1.1. Undulating Fin Kinematic Model

Describing the motion of the undulating fin in the fin coordinate system $O_{u}-X_{u}Y_{u}Z_{u}$, let any point on the fin surface be represented as $P_{1}(x_{u},y_{u},z_{u})$, the initial position of the undulating fin is as described in the following:

(11) $$\left\{\begin{array}{l} x_{u}=s\\ y_{u}=r\cdot \cos\left[\theta_{m}\sin\left(\delta_{1}\cdot 2\pi f_{1}+\frac{2\pi }{\lambda }s\right)\right]\\ z_{u}=r\cdot \sin\left[\theta_{m}\sin\left(\delta_{1}\cdot 2\pi f_{1}+\frac{2\pi }{\lambda }s\right)\right] \end{array}\right.$$

where *s* is the natural coordinate in the baseline direction of the undulating fin, *r* is the natural coordinate in the fin direction, *f*_1_ are the undulating frequencies, respectively, λ is the wavelength, *θ_m_* is the wave amplitude, and *δ*_1_ represents the wave transmission direction.

The position of the wave fin coordinate system’s origin relative to the robot coordinate system is denoted as (*a*, *b*, *c*). The angles between the undulating fin and the body are *φ*_1_. According to the rotation relationship, the coordinates of point within the geographical system are determined as follows:

(12) $$\left[\begin{array}{c} x_{e}\\ y_{e}\\ z_{e} \end{array}\right]=J_{1}(\eta )\left[\begin{array}{c} x_{u}+a\\ y_{u}\cos\phi_{1}-z_{u}\sin\phi_{1}+b\\ y_{u}\sin\phi_{1}+z_{u}\cos\phi_{1}+c \end{array}\right]+\left[\begin{array}{c} X\\ Y\\ Z \end{array}\right]$$

Here, *X*, *Y*, and *Z* represent the distances from the robot’s coordinate system origin to the world coordinate system’s *X*, *Y*, and *Z* axes, respectively.

#### 3.1.2. Flapping Wing Kinematic Model

**Figure 7:**
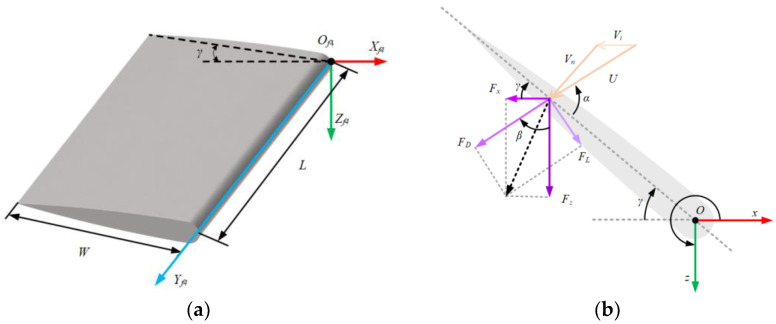
Flapping wing kinematic model. (**a**) Flapping wing 3D kinematic model; (**b**) Forces, flow velocity and angle on the flapping wing.

When the underwater robot is moving in the water and driven solely by the flapping wings, a single wing structure is shown in Figure 7a, where the wing length is *L* and the width is *W*. The forces acting on the wing are illustrated in Figure 7b. The flapping wing swings around its short edge in the *xz* plane about point O*_f_*, with the water flow generating a normal velocity perpendicular to the fin surface at the point of contact. During the flapping motion, the wing displaces water in the region it traverses, which leads to a drop in fluid pressure in that region. The surrounding fluid rapidly fills the space once occupied by the fin, generating an inflow velocity [22,23]. The normal velocity and the inflow velocity are combined through vector addition to form the total water flow velocity *U*.

The flapping wing propellers are placed on both sides of the robot, with the wings rotating around the rotational axis. The equation of motion is as follows:

(13) $$\gamma (t)=\gamma_{0}\sin(2\pi f_{p}t)+\gamma_{\mathrm{bias}}$$

In this case, $\gamma_{0}$ denotes the amplitude of the flapping wing motion, $f_{p}$ is the flapping frequency, and $\gamma_{\mathrm{bias}}$ represents the equilibrium bias angle.

During the flapping motion of the wing, α denotes the angle between the water flow velocity U and the flapping wing (angle of attack); *β* is the angle between the direction of drag and the z-axis; *γ* represents the angle between the flapping wing and the z-axis. Based on the literature [24] and as shown in Figure 7b, the relationship between α, *β*, and *γ* can be expressed as follows:

(14) $$\gamma (t)=\alpha +\beta -\frac{\pi }{2}$$

Taking the right rear flapping wing as an example, as shown in Figure 7a, assume that the position of any point on the right rear flapping wing in the flapping–wing coordinate system is $O_{4}(x_{f4},y_{f4},z_{f4})$. Then, the coordinate system of the flapping wing is transformed into the coordinate system of the robot body as follows:

(15) $$\left\{\begin{array}{l} x_{f4}=-w\cos\gamma ,0\leq w\leq W\\ y_{f4}=l,0\leq l\leq L\\ z_{f4}=w\sin\gamma ,0\leq w\leq W \end{array}\right.$$

Here, *w* and *l* represent the width and length of a specific point on the wing in the wing coordinate system.

Let the origin *O_f_*_4_ of the right rear flapping wing’s coordinate system, expressed in the robot’s coordinate system, be given by the position vector $\left(a_{f4},b_{f4},c_{f4}\right)$. The angle between the flapping wing and the body is *φ*_2_. If the coordinates of an arbitrary point *P*_4_ on the right rear flapping wing, in its own coordinate system, are represented by the vector $P_{4}(x_{b4},y_{b4},z_{b4})$, then the coordinates of *P*_4_ in the body coordinate system can be determined by the following transformation:

(16) $$\left[\begin{array}{c} x_{b4}\\ y_{b4}\\ z_{b4} \end{array}\right]=\left[\begin{array}{ccc} \cos\varphi_{2} & 0 & -\sin\varphi_{2}\\ 0 & 1 & 0\\ \sin\varphi_{2} & 0 & \cos\varphi_{2} \end{array}\right]\left[\begin{array}{c} x_{f4}\\ y_{f4}\\ z_{f4} \end{array}\right]+\left[\begin{array}{c} a_{f4}\\ b_{f4}\\ c_{f4} \end{array}\right]$$

Finally, the coordinates of $P_{4}(x_{e4},y_{e4},z_{e4})$ in the robot coordinate system in the world coordinate system are as follows:

(17) $$\left[\begin{array}{c} x_{e4}\\ y_{e4}\\ z_{e4} \end{array}\right]=J_{1}(\eta )\left[\begin{array}{c} x_{b4}\\ y_{b4}\\ z_{b4} \end{array}\right]+\left[\begin{array}{c} X\\ Y\\ Z \end{array}\right]$$

Here, *X*, *Y*, and *Z* represent the distances from the robot’s coordinate system origin to the world coordinate system’s *X*, *Y*, and *Z* axes, respectively.

### 3.2. Hydrodynamic Modeling of Composite-Driven Underwater Robots

#### 3.2.1. Flapping Wing Dynamics Model

The flapping wing generates lift *F_L_* and drag *F_D_* during the flapping motion. The drag *F_D_* is in the same direction as the water flow velocity U, and the lift *F_L_* is perpendicular to the drag, as shown in Figure 7b. The angle of attack α represents the position of the blade relative to the water flow. The expressions for lift *F_L_*, drag *F_D_*, and the resultant force *F_HD_* are given as follows [25,26,27]:

(18) $$F_{L}=\frac{\rho U^{2}SC_{Lmax}\sin(2\alpha )}{2}$$

(19) $$F_{D}=\frac{\rho U^{2}SC_{Dmax}(1-\cos(2\alpha ))}{2}$$

(20) $$F_{H}=\sqrt{F_{D}^{2}+F_{L}^{2}}$$

In this case, *ρ* is the water density, *S* is the equivalent area of the flapping wing’s stroke plane, and *C_Dmax_* and *C_Lmax_* represent the maximum drag coefficient and maximum lift coefficient of the flapping wing, respectively.

The lift *F_L_* and drag *F_D_* can be transformed into forces along the coordinate axes:

(21) $$\left\{\begin{array}{l} F_{x}=F_{Di}\sin\beta_{i}+F_{Li}\cos\beta_{i}\\ F_{z}=-F_{Li}\sin\beta_{i}+F_{Di}\cos\beta_{i} \end{array}\right.$$

The total thrust of the four flapping wings and its components along the *x* and *y* axes are shown as follows:

(22) $$\left\{\begin{array}{l} F_{x}^{’}=\sum_{i=1}^{4}(F_{Di}*\mathrm{Sin}\beta_{i}+F_{Li}*\cos\beta_{i})\\ F_{z}^{’}=\sum_{i=1}^{4}(-F_{Li}*\sin\beta_{i}+F_{Di}*\cos\beta_{i}) \end{array}\right.$$

The total thrust torque around the *x*, *y*, and *z* axes is shown as follows:

(23) $$\left\{\begin{array}{l} M_{x}=\sum_{i=1}^{4}\left(y_{i}\left(-F_{Li}*\sin\beta_{i}+F_{Di}*\cos\beta_{i}\right)\right)\\ M_{y}=\sum_{i=1}^{4}\left(x_{i}(-F_{Li}*\sin\beta_{i}+F_{Di}*\cos\beta_{i})+z_{i}(F_{Di}*\mathrm{Sin}\beta_{i}+F_{Li}*\cos\beta_{i})\right)\\ M_{z}=\sum_{i=1}^{4}\left(-y_{i}(F_{Di}*\sin\beta_{i}+F_{Li}*\cos\beta_{i})\right) \end{array}\right.$$

In this case, (*x_i_*,*y_i_*,*z_i_*) denotes the position coordinates of the *i*-th flapping wing in the body coordinate system.

The dynamic analysis of the undulating fin is thoroughly detailed in Section 2.3 of reference [28] and is therefore not reiterated herein.

#### 3.2.2. Hydrodynamic Modeling of the Robot

In the local coordinate system of the underwater robot, the external forces *F* and moments *M* are represented as follows [29]:

(24) $$\left\{\begin{array}{l} F={\left(F_{x},F_{y},F_{z}\right)}^{T}\\ M={\left(M_{x},M_{y},M_{z}\right)}^{T} \end{array}\right.$$

The dynamics of the robot’s motion are described by the following equations:

(25) $$\left\{\begin{array}{l} F=m\frac{dv}{dt}+\omega \times mv+F_{\mathrm{drag}}(v)+F_{\mathrm{ext}}\\ M=J\frac{d\omega }{dt}+\omega \times J\omega +M_{\mathrm{drag}}(\omega )+M_{\mathrm{ext}} \end{array}\right.$$

where *m* is the mass of the robot. *J* is the inertia tensor of the robot. *v* is the linear velocity vector of the robot. *ω* is the angular velocity vector of the robot. $F_{\mathrm{drag}}(v)$ is the drag force, which depends on the velocity *v*. $F_{\mathrm{ext}}$ is the external force acting on the robot. $M_{\mathrm{drag}}(\omega )$ is the drag moment, which depends on the angular velocity *w*. $M_{\mathrm{ext}}$ is the external moment acting on the robot.

Substituting the revised force and moment equations yields the updated velocity and angular velocity components. The components of acceleration are obtained from the equations of force and moment.

(26) $$\left\{\begin{array}{l} \dot{u}=vr-wq+\frac{F_{x}}{m}+\frac{F_{\mathrm{drag}x}(v)}{m}+\frac{F_{\mathrm{ext}x}}{m}\\ \dot{v}=-ur+wp+\frac{F_{y}}{m}+\frac{F_{\mathrm{drag}y}(v)}{m}+\frac{F_{\mathrm{ext}y}}{m}\\ \dot{w}=-uq+vp+\frac{F_{z}}{m}+\frac{F_{\mathrm{drag}z}(v)}{m}+\frac{F_{\mathrm{ext}z}}{m} \end{array}\right.$$

For angular velocity dynamics,

(27) $$\left\{\begin{array}{l} \dot{p}=qr(J_{y}-J_{z})+\frac{1}{J_{x}}M_{x}+\frac{1}{J_{x}}M_{\mathrm{drag}x}(\omega )+\frac{1}{J_{x}}M_{\mathrm{ext}x}\\ \dot{q}=pr(J_{z}-J_{x})+\frac{1}{I_{y}}M_{y}+\frac{1}{J_{y}}M_{\mathrm{drag}y}(\omega )+\frac{1}{J_{y}}M_{\mathrm{ext}y}\\ \dot{r}=pq(J_{x}-J_{y})+\frac{1}{J_{z}}M_{z}+\frac{1}{J_{z}}M_{\mathrm{drag}z}(\omega )+\frac{1}{J_{z}}M_{\mathrm{ext}z} \end{array}\right.$$

where $J_{x}$, $J_{y}$, and $J_{z}$ are the moments of inertia of the robot about the *x*, *y*, and *z* axes, respectively. $M_{x}$, $M_{y}$, and $M_{z}$ are the moments about the corresponding axes. $M_{\mathrm{drag}x}(\omega )$, $M_{\mathrm{drag}y}(\omega )$, and $M_{\mathrm{drag}z}(\omega )$ are torques generated by aerodynamic drag or damping. $M_{\mathrm{ext}x}$, $M_{\mathrm{ext}y}$, and $M_{\mathrm{ext}z}$ are other external torques.

### 3.3. Simulation Model

**Figure 8:**
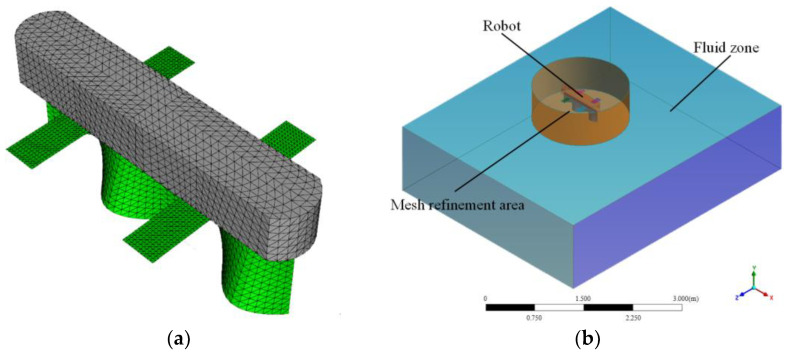
CFD simulation model: (**a**) the mesh model of the robot; (**b**) the calculation zone.

Computational fluid dynamics (CFD) simulations were performed to assess the feasibility of the robot’s design and its motion capabilities by analyzing the forces and motion within the flow field under different control inputs [30,31]. To enhance grid division efficiency and accelerate the simulation, the robot’s body was chamfered, and the flapping wings were reduced to a planar model. Furthermore, the actual thickness of the robot’s undulating fin is 2 mm. Compared to the overall characteristic length of the robot, this thickness is essentially negligible. Therefore, in the simulation, a rational approximation was made by simplifying it to a 3D surface with zero thickness. Figure 8a illustrates the establishment of the simulation model, while Figure 8b depicts the fluid domain. The outer fluid region was segmented into a coarse mesh, whereas the robot and its surrounding areas were refined using a finer mesh. The simulation parameters of the robot are presented in Table 1, derived from the design parameters of the physical prototype.

This study employs computational fluid dynamics (CFD) simulation software (version 2021) to solve the Reynolds-Averaged Navier–Stokes (RANS) equations. The Shear Stress Transport (SST) k-ω model was selected to handle large deformation flows, as it is also suitable for complex flow phenomena near the undulating fins and the main body wall. The inlet and outlet boundaries were defined as velocity inlet and pressure outlet, respectively, while internal regions were treated as internal boundaries, and the fluid domain walls were set as viscous walls. Under quiescent water conditions and to meet convergence criteria, the inlet velocity, time step, maximum iterations per time step, and convergence criterion were set to 0.001 m/s, 0.002 s, 20, and 10^−5^, respectively. The pressure–velocity coupling was handled using a second-order upwind scheme to ensure simulation accuracy.

## 4. Hydrodynamic Simulation and Experiments

### 4.1. Robot Prototype

**Figure 9:**
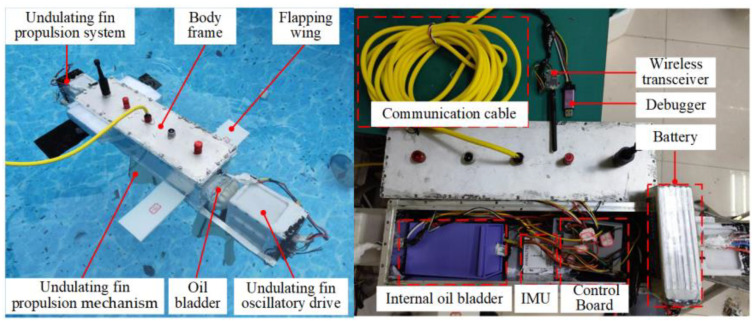
Multi-modal composite-driven robot prototype.

A robot prototype was developed to conduct physical experiments for verifying the hydrodynamic performance in the CFD simulation. Figure 9 illustrates that the prototype comprises six components: the undulating fin deflection module, the body, the flapping wings, the undulating fin drive module, the external oil bladder, and the undulating fins. The body contains integrated components including a battery, control board, gyroscope, and internal oil bladder. The exterior features communication wires, a wireless receiver, and a debugger. The robot possesses a rectangular structure, and in its initial state, buoyancy marginally exceeds gravitational force. The buoyancy adjustment mechanism serves to equilibrate buoyancy and gravitational forces. The robot’s exterior comprises aluminum alloy, while the undulating fins consist of flexible fan-shaped silicone. The flapping wings are fabricated through 3D printing with PLA material. Table 2 presents the primary parameters of the robot prototype.

**Figure 10:**
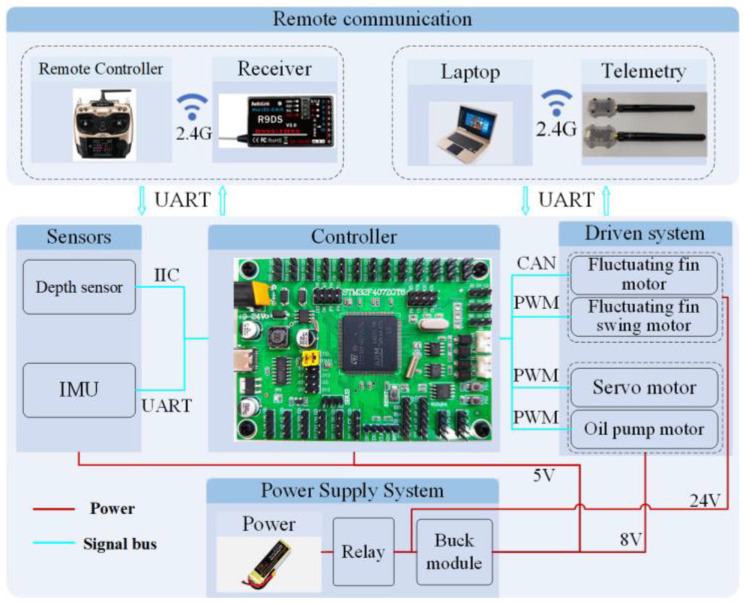
Control system schematic.

The multi-modal control system of the composite-driven underwater robot comprises the main control system, sensor system, communication system, drive system, and power system, with the objective of ensuring stable underwater communication and precise motion control. The primary control system employs the STM32F407 series chip from STMicroelectronics (Italy), which is based on the ARM core and operates at a clock frequency of up to 168 MHz, as illustrated in Figure 10. The control system unit executes multiple functions: 

Wireless communication is facilitated through a 2.4G wireless data communication device and data transmission module, which receive control commands from a remote handheld controller or ground station. The commands encompass motion modes such as forward/backward, ascent/descent, turning, and gliding, as well as control variables including position and movement speed.

Sensor data transmitted from the controller is accessed via the serial bus, followed by signal filtering and data fusion processes.

A closed-loop control system is implemented for the drive motors via an inner PID controller. Subsequently, an outer PID controller, in conjunction with an IMU sensor, calculates the discrepancy between target and current values. This enables regulation of the flapping speed of the four wing motors, the rotational speed of the undulating fin motors, the swing angle of the undulating fin swing motors, and the intake and discharge of hydraulic oil through the oil bladder motor and reversing motor. The composite-driven underwater robot’s status is transmitted to the host computer via the data transmission module, with real-time data presented through the ground station software.

The control methods for the composite-driven underwater robot consist of operation through a handheld remote device and a laptop, with the latter being prioritized. The handheld remote control modifies the robot’s motion mode and speed through buttons, dip switches, and joysticks, rendering it appropriate for visual operation in underwater tasks. The control of the laptop is facilitated by a pair of wireless data transmission modules, which allow for precise parameter adjustments and data storage, functioning as the primary control method in experiments.

### 4.2. Simulation Result

#### 4.2.1. Undulating Motion

**Figure 11:**
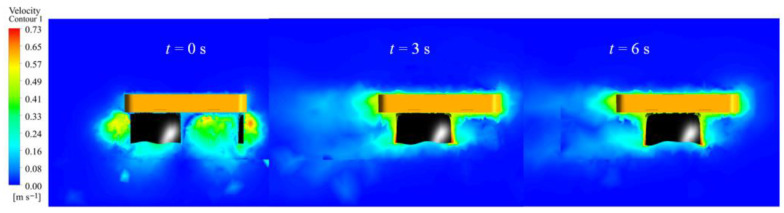
Velocity cloud image sequence of the robot in straight motion driven by the undulating fin mode.

**Figure 12:**
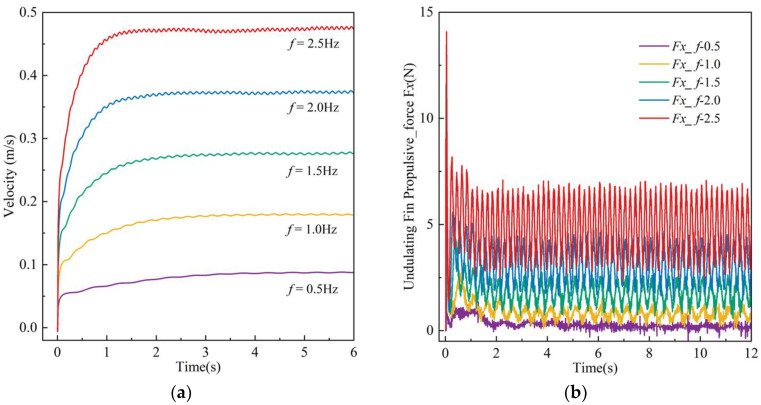
Simulation results of the robot in linear motion by the undulating fin mode: (**a**) velocity and displacement; (**b**) thrust force.

The robot’s linear motion in the fluid was simulated based on a fin frequency range of 0.5–2.5 Hz. Figure 11 illustrates the velocity contour sequence of the robot at a frequency of 2.5 Hz, with various colors indicating the velocity distribution within the flow field, and the animation video is available in the Supplementary Materials. The findings indicate that the robot’s propulsion direction is forward, with no lateral displacement or steering observed during linear motion. Figure 12 illustrates the robot’s velocity curve, depicting an initial acceleration phase followed by the attainment of maximum speed and subsequent maintenance of a constant speed phase. At an undulating frequency of 2.5 Hz, the robot achieves a speed of 0.47 m/s.

Figure 12b illustrates the thrust exerted on the robot during motion. The simulation results indicate that the thrust generated by the undulating fin quickly attains its maximum during the initial phase, subsequently declining as the robot’s speed escalates. This phenomenon results from the viscous drag exerted by the stationary water on the oscillating fin during startup. Zhou and Yin’s research [32] has validated this trend. During the steady phase, the thrust produced by the undulating fin varies around its mean value, demonstrating that the oscillating fin’s motion can consistently generate forward thrust to advance the robot. An increase in undulating frequency correlates with a rise in vibration frequency, leading to an enhancement in fluid force.

**Figure 13:**
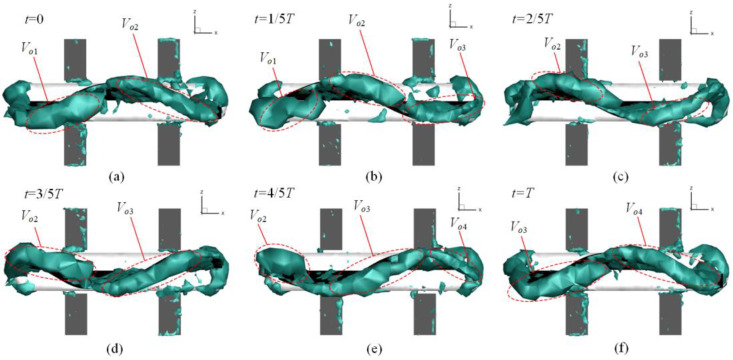
Evolution of vortex structures during one period of wave motion, based on the Ω-criterion, iso-surface of Ω = 0.52: (**a**) *t* = 0; (**b**) *t* = 1/5*T*; (**c**) *t* = 2/5*T*; (**d**) *t* = 3/5*T*; (**e**) *t* = 4/5*T*; (**f**) *t* = *T*. Within a single cycle, a central jet forms along the outer edge of the fin and is subsequently segmented into multiple vortex regions. These vortices, numbered *V_o_*_1_ to *V_o_*_4_ from the trailing edge to the leading edge, exhibit distinct generation and shedding patterns. *V_o_*_1_ is the first to be generated and shed, while *V_o_*_4_ is the last. Specifically, *V_o_*_1_ and *V_o_*_3_ are located on the ventral side of the fin, and *V_o_*_2_ and *V_o_*_4_ are situated on the dorsal side. During the interval from *t* = 0 to *t* = 2/5*T*, *V_o_*_1_ detaches from the trailing edge, and *V_o_*_3_ is generated on the ventral side of the leading edge. Meanwhile, *V_o_*_2_ continues to develop along the direction of wave propagation. Between *t* = 1/5*T* and *t* = 2/5*T*, *V_o_*_1_ completely separates and transforms into the wake. From *t* = 3/5*T* to *t* = *T*, *V_o_*_2_ detaches from the trailing edge, and *V_o_*_4_ is generated on the dorsal side of the leading edge. *V_o_*_3_ then continues to develop along the direction of wave propagation. Between *t* = 4/5*T* and *t* = *T*, *V_o_*_2_ completely separates and transforms into the wake. The evolution of these vortex structures indicates that vortex shedding into the wake contributes to the thrust generated by the undulating fin.

According to [32], the vortex shedding phenomenon downstream of the caudal fin represents the generation of forward thrust. Concurrently, a central jet forms along the direction of propulsion, and these two mechanisms collectively dominate the force generation and kinematic patterns of the robot. Figure 13 illustrates the evolution of vortex structures, visualized on an iso-surface, under the undulating fin locomotion mode when the robot achieves a stable moving state. The criterion value for Ω was set to 0.52, with the 3D view oriented towards the ventral side of the undulating fin.

#### 4.2.2. Flapping Motion

(1) Linear Motion

**Figure 14:**
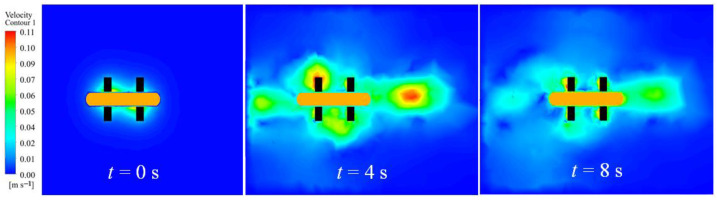
Sequence of velocity contour plots of the robot in flapping mode during straight motion.

A simulation analysis was performed to evaluate the performance of the robot utilizing flapping wing motion in the turning mode. The flapping frequency was established at 1–5 Hz, with corresponding flapping amplitudes of 15°, 20°, and 25°. The simulation results indicate that with a flapping amplitude of 25° and a flapping frequency of 4 Hz, the velocity contour sequence is presented in Figure 14 (the animation video can be found in the Supplementary Materials). Various colors denote the velocity distribution within the flow field. The findings indicate that the robot produces forward thrust, resulting in linear motion devoid of lateral or rotational movement.

**Figure 15:**
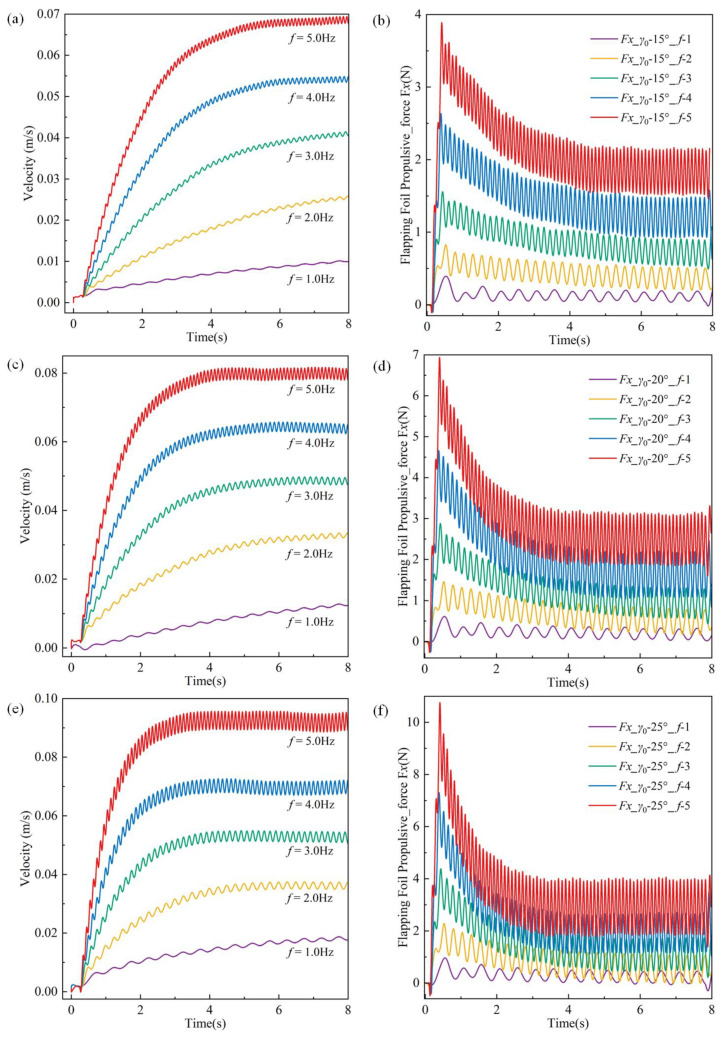
Simulation speed and corresponding thrust variation of the robot in flapping wing mode under different amplitudes and frequencies. (**a**) Simulation speed, 15°; (**b**) Simulated thrust, 15°; (**c**) Simulation speed, 20°; (**d**) Simulated thrust, 20°; (**e**) Simulation speed, 25°; (**f**) Simulation speed, 25°.

Figure 15 presents the simulated velocity and thrust variations of the underwater robot’s flapping wing motion across different flapping amplitudes (15°, 20°, 25°) and frequencies (1–5 Hz). Figure 15a,c,e illustrate the robot’s velocity variation over time at amplitudes of 15°, 20°, and 25°, respectively. At a constant flapping frequency, an increase in amplitude results in a corresponding increase in the robot’s velocity. The variation in speed changes across different frequencies is markedly significant. An increase in frequency correlates with a higher rate of speed increase. 

Figure 15b,d,f illustrate the total thrust variation of the four flapping wings across various flapping amplitudes and their corresponding frequencies. With an increase in frequency, thrust initially rises rapidly before gradually stabilizing. Increased frequency correlates with a greater fluid force exerted on the oscillating fin. The peak thrust varies with different flapping amplitudes; higher amplitudes yield greater initial peak thrust and a reduced time to achieve steady velocity.

**Figure 16:**
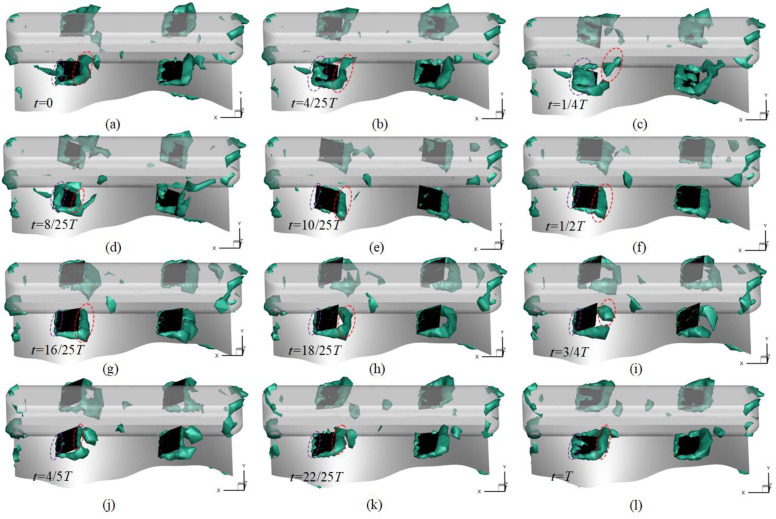
Evolution of vortex structures during one period of flapping motion, based on the Ω-criterion, iso-surface of Ω = 0.57: (**a**) *t* = 0; (**b**) *t* = 4/25*T*; (**c**) *t* = 1/4*T*; (**d**) *t* = 8/25*T*; (**e**) *t* = 10/25*T*; (**f**) *t* = 1/2*T*; (**g**) *t* = 16/25*T*; (**h**) *t* = 18/25*T*; (**i**) *t* = 3/4*T*; (**j**) *t* = 4/5*T*; (**k**) *t* = 22/25*T*; (**l**) *t* = *T*.

Figure 16 presents the evolution of vortex structures on an iso-surface for a robot operating in steady locomotion, with Ω = 0.57. The robot translates along the positive x-direction in a four-flap flapping mode; all four flaps share identical frequency and amplitude. During *t* = 0 to 1/4*T*, the flaps move downward from the horizontal to their maximum stroke. Leading-edge vortices form first and develop rearward along the flap surface toward the trailing edge; at *t* = 1/4 *T* these vortices reach maximum strength and are nearly fully shed. From *t* = 1/4 *T* to 3/4 *T*, the flaps move upward to their opposite maximum stroke; leading-edge vortices again appear first and grow rearward, beginning to shed near *t* = 1/2 *T* and completing shedding by *t* = 3/4 *T*. From *t* = 3/4 *T* to *T*, the flaps return downward to the horizontal; leading-edge vortices form and propagate rearward until they begin to shed at t = *T*. Over a single cycle, the observed vortex dynamics indicate that flapping generates vortex regions at the fin edges, with trailing-edge vortices larger than leading-edge vortices; the trailing-edge vortices thus make a greater contribution to thrust generation.

(2) Steering Motion

**Figure 17:**
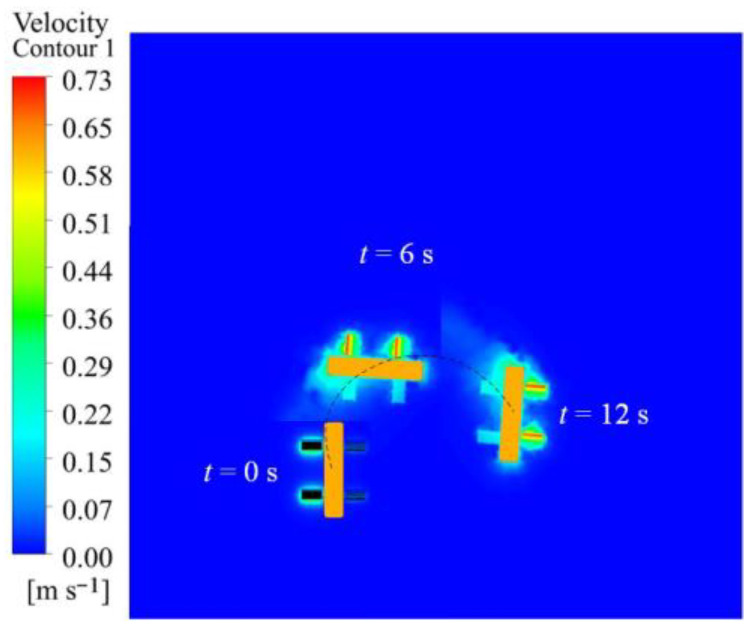
Velocity cloud image sequence of the robot in steering motion simulation under different wing amplitudes.

A simulation was conducted to examine the impact of varying flapping amplitudes on the robot’s motion efficacy in the turning mode, utilizing solely flapping wing motion. The flapping frequency for both the left and right wings is established at 3 Hz, with left wing amplitudes of 30°, 40°, 50°, and 60°, while the right wing amplitude remains constant at 10°. The simulation results demonstrate that the robot deviates to the right while moving forward, with the motion posture and spatial position illustrated in Figure 17, displaying the velocity contour with a left wing amplitude of 50°. The animation video can be found in the Supplementary Materials. The results demonstrate that the flow velocity is initially minimal, and as the flapping motion advances, the robot incrementally accelerates from the fluid and attains a stable condition.

**Figure 18:**
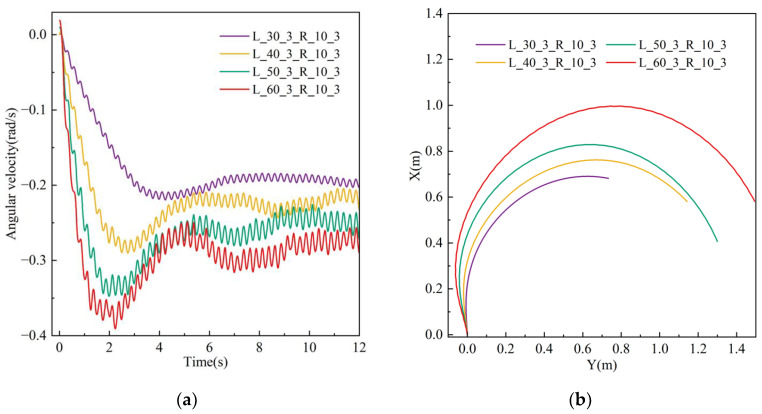
Simulation results of the robot in steering motion under different wing amplitudes: (**a**) angular velocity; (**b**) displacement.

The robot’s rotational angular velocity in the XZ plane is displayed in Figure 18a. As the flapping amplitude grows, the robot’s rotational velocity similarly increases, with a rapid rise in the initial phase, followed by stabilization. Figure 18b illustrates the robot’s movement trajectory, depicting its continuous turning motion around a circular arc in the XZ plane. The tangent direction at any point on the trajectory aligns with the direction of the robot’s forward velocity.

**Figure 19:**
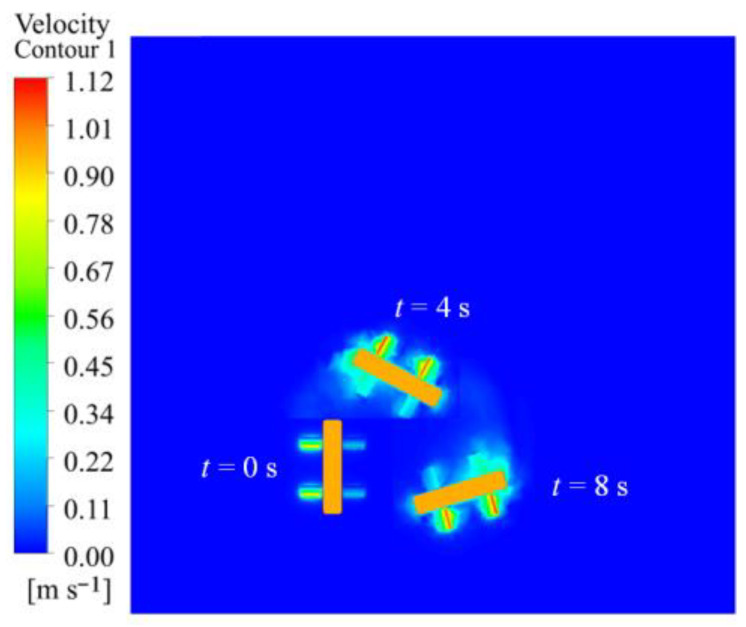
Velocity cloud image sequence of the robot in steering motion simulation under different wing frequencies.

A simulation was conducted to assess the impact of varying flapping frequencies on the robot’s motion performance while turning, utilizing solely flapping wing motion. The amplitudes of the left and right flapping wings are both established at 50°, with the left wing frequencies designated at 2 Hz, 3 Hz, 4 Hz, and 5 Hz, while the right wing frequency remains constant at 1 Hz. The simulation results demonstrate that the robot deviates to the right when moving forward, with the motion posture and spatial position at 4 Hz illustrated in Figure 19. The findings demonstrate that the flow velocity is initially little, and as the flapping motion persists, the robot’s speed progressively escalates and stabilizes.

**Figure 20:**
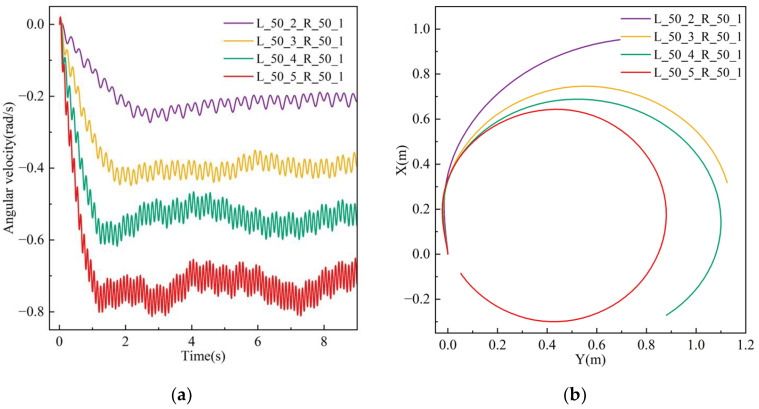
Simulation results of the robot in steering motion under different wing frequencies: (**a**) angular velocity; (**b**) displacement.

Figure 20a illustrates the robot’s rotating angular velocity in the XZ plane. As the flapping frequency escalates, the robot’s angular velocity correspondingly increases, initially surging fast before stabilizing. Figure 20b illustrates the motion trajectory, whereby the robot executes a turning motion along a circular arc in the XZ plane. The tangent vector at any point on the trajectory aligns with the robot’s forward velocity vector. The animation video can be found in the Supplementary Materials.

In conclusion, we conducted simulation assessments of the robot’s turning behavior at various flapping amplitudes and frequencies in the flapping wing mode. The findings indicate that an increase in flapping amplitude correlates with a bigger turning radius of the robot, whereas a higher flapping frequency is associated with a reduced turning radius.

### 4.3. Experiment Validation

This article conducted trials on the robot’s forward motion, turning, and buoyancy-sinking action based on the designed prototype. The studies on undulating fin and flapping wing propulsion assessed the effect of varying undulating frequencies on the robot’s forward velocity, and the propulsion speed performance was validated. The buoyancy-sinking function experiments examined the impact of the oil bladder and flapping wings on the robot’s buoyancy-sinking dynamics. The experimental results were documented and stored in two formats: a high-speed camera (GoPro) was positioned in front of the pool to document the robot’s movement during each test. Secondly, the lower-level controller of the composite-driven underwater robot transmits real-time data (including attitude angles, depth, etc.) to the laptop at a frequency of 50 Hz, while a proprietary upper-level computer is employed for real-time visualization and an archive of historical data.

#### 4.3.1. Linear Motion

- Undulating Fin Linear Motion Test

**Figure 21:**
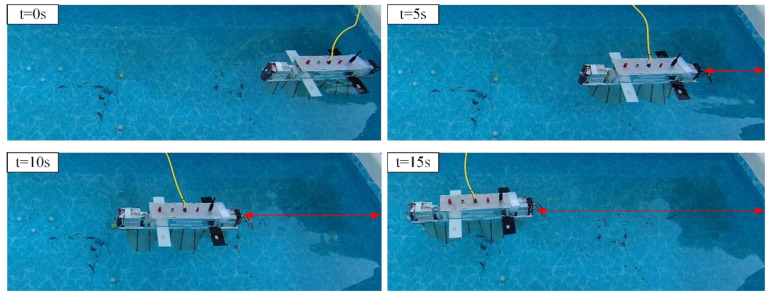
Undulating fin linear motion test.

**Figure 22:**
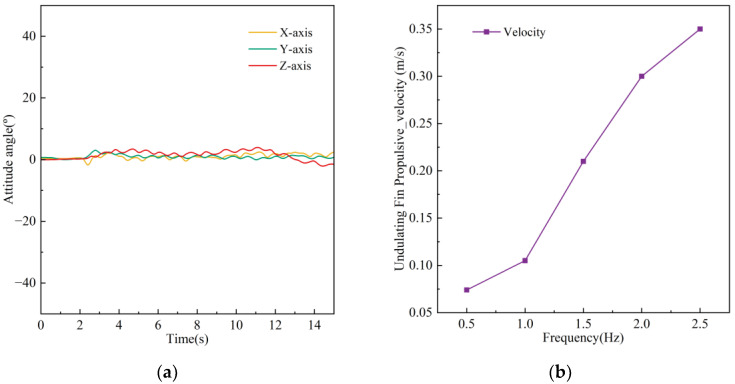
Robot undulating motion data. (**a**) Change in robot pose. (**b**) Relationship between undulating speed and frequency.

In the experiment involving undulating fin forward motion, the robot initiates movement from the right side of the pool, with the undulating fin oscillating at a frequency of 2 Hz. The undulating fin linear motion experiments video can be found in the Supplementary Materials. It shifts to the left and halts upon reaching the terminus. Figure 21 illustrates the sequence of images depicting the robot’s forward movement, while Figure 22a displays the temporal variation of the attitude angles across each axis. The experimental results indicate that, under undulating fin propulsion, the robot can advance smoothly, maintaining the attitude angle deviation of each axis within 5°. We conducted seven repeated straight-line locomotion tests at each undulation frequency, calculated the mean forward speed, and thereby evaluated the effect of undulation frequency on forward velocity. The results are shown in Figure 22b; at an undulation frequency of 2.5 Hz, the robot attained a peak speed of 0.35 m/s.

- Flapping Wing Linear Motion Test

**Figure 23:**
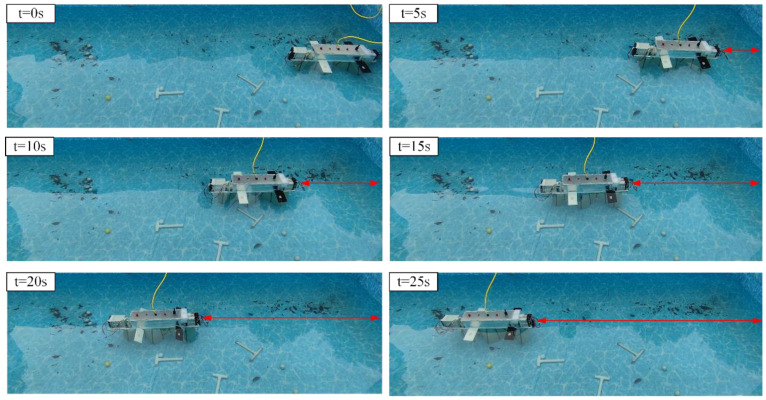
Flapping wing linear motion test.

In addition to attitude control, the flapping wings may produce thrust, rendering them appropriate for low-speed cruising and precise attitude regulation situations. The flapping wing linear motion experiments video can be found in the Supplementary Materials. In the flapping wing linear motion experiment, the robot initiates from the right side of the pool, flapping its wings at an amplitude of 40° and a frequency of 5 Hz, progressing to the left and decelerating upon reaching the endpoint. Figure 23 illustrates the series of images pertaining to this process.

**Figure 24:**
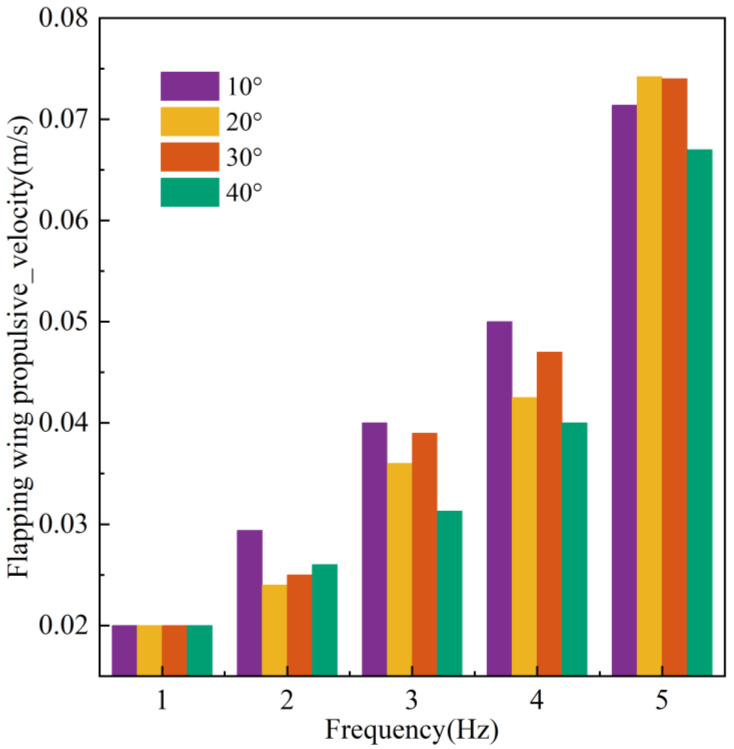
The relationship between flapping speed, amplitude, and frequency.

To investigate the influence of flapping frequency on the robot’s forward velocity, seven replicate experiments were conducted at each frequency, yielding the average flapping speed. Figure 24 analyzes the velocity characteristics of the flapping wing drive across different frequencies and amplitudes. Generally, the robot’s forward velocity is directly proportional to the flapping drive frequency. At the same frequency, the highest velocity was achieved with an amplitude of 20°, while the lowest was observed at an amplitude of 80°. This phenomenon is attributed to the smaller vertical force and lower drag generated during small-angle flapping, contrasted with the larger vertical force and increased drag at large angles, compounded by reduced servo motor response speed at high frequencies and large displacements.

#### 4.3.2. Rotation Motion

- Undulating Fin Turning Motion Test

**Figure 25:**
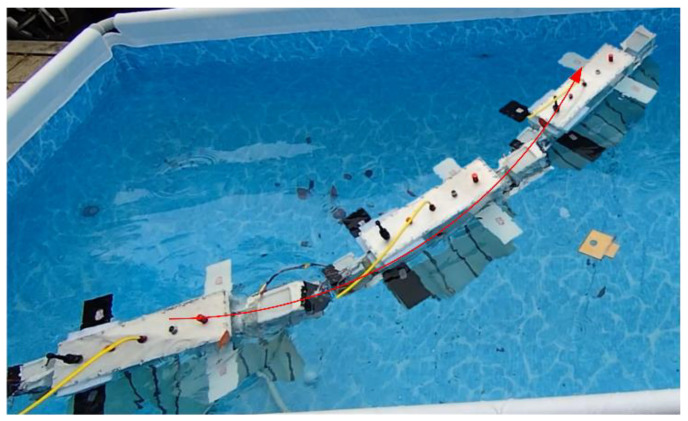
Undulating fin turning test.

In the undulating fin turning experiment, as shown in Figure 25, the undulating fin oscillates with a 60° amplitude and a frequency of 1 Hz, enabling the robot to achieve a smooth turn with an average turning speed of about 5°/s. The undulating fin turning motion video can be found in the Supplementary Materials.

- Flapping Wing Turning Motion Test

**Figure 26:**
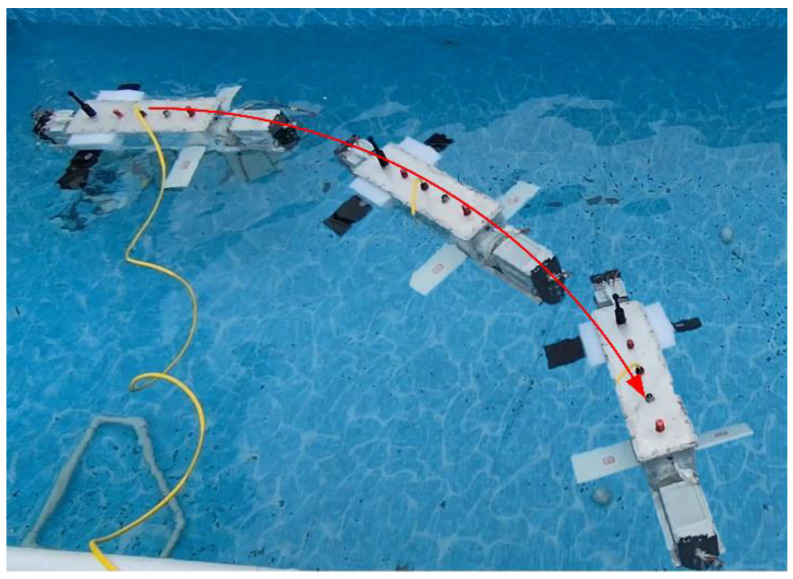
Flapping wing turning motion test.

In the flapping wing turning experiment, as shown in Figure 26, the right wing flaps with a 10° amplitude at a frequency of 3 Hz, whereas the left wing flaps with a 30° amplitude at the same frequency of 3 Hz. The robot rotates at an average angular velocity of 2.5°/s. The flapping wing turning motion video can be found in the Supplementary Materials.

#### 4.3.3. Buoyancy and Submersion Movement

**Figure 27:**
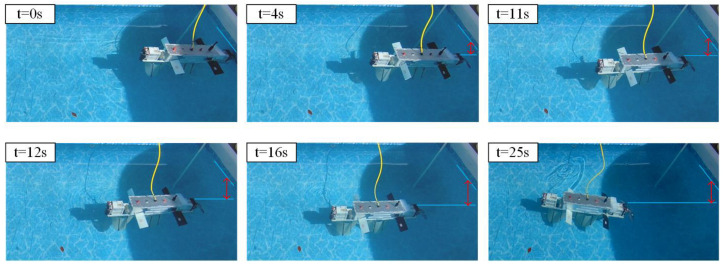
Buoyancy movement experiment.

As shown in Figure 27, to validate the robot’s buoyancy in water, a dynamic buoyancy experiment was conducted, encompassing sinking and rising phases. At the start, the robot transfers hydraulic oil from an external oil bladder to an internal oil bladder using an oil pump, reducing the overall volume and buoyancy, which leads to sinking. During the sinking phase, the flapping wings actuate cyclically in the first quadrant, producing horizontal and vertical downward movements and attitude adjustments. Subsequently, the oil is transferred back from the internal bladder to the external bladder, increasing the overall volume and buoyancy to achieve ascent. During the rising phase, the wings actuate cyclically in the fourth quadrant, producing horizontal and vertical upward movements and attitude adjustments. The experimental results demonstrate the effectiveness of the control strategy proposed in this study during the sinking and rising processes.

## 5. Discussion

The integrated undulatory fin and flapping foil composite actuation mode proposed in this study achieves a logical equilibrium between biomimetic principles and engineering application in its design. While this structure deviates from a direct imitation of a singular biological prototype, such as the knifefish, this represents a deliberate engineering trade-off: it retains the advantages of undulatory fins in low-to-medium speed propulsion and environmental adaptability, while simultaneously acquiring vector thrust control through independent flapping foil units, thereby extending the capacity for rapid attitude adjustment. Knifefish primarily achieve turning through non-routine movements of asymmetric fins, exhibiting excellent stealth and maneuverability at low-to-medium speeds and in complex aquatic environments. However, they possess inherent limitations in seabed resource exploration and rapid attitude changes. By coupling these two forms of actuation, this paper preserves the deep biological mechanisms while introducing engineered implementation methods, thereby achieving broad adaptability to multiple operating conditions and enhancing task execution capabilities. The undulatory fins shoulder the advantages of stable propulsion at low-to-medium speeds and environmental coupling, while the flapping foil units provide flexible vector output, rendering the system more responsive, robust, and controllable in tasks such as deep-sea exploration and resource surveying. This design is not a simple replication of biological morphology but rather a functional reconstruction and performance optimization based on biological locomotion mechanisms, aiming to achieve broader operational adaptability and task execution capabilities than a singular biological prototype.

Leveraging the high maneuverability, controllable vector thrust, low-noise propulsion, and optimized energy consumption characteristics afforded by the coupling of undulatory fins and flapping foils, the multimodal composite actuation platform designed in this study exhibits significant adaptability and advantages across a variety of practical application scenarios, specifically the following:

- In deep-sea and complex terrain exploration, the system can achieve steady-state cruising and environmental coupling via the undulatory fins. For localized detection, sample collection, and pipeline inspection tasks requiring fine attitude adjustment or rapid maneuvers, the flapping foil units can provide instantaneous vector thrust and highly responsive attitude control.
- In oceanographic observation and ecological monitoring, the low-noise propulsion method is conducive to minimizing disturbance to marine life, thereby enhancing the veracity of sensor data.
- In disaster search-and-rescue and near-shore patrol scenarios, the platform’s agile locomotion and energy efficiency advantages can improve mission response times and endurance efficiency.
- Furthermore, this design facilitates the integration of a variety of sensors and operational modules, making it suitable for joint exploration, long-term persistent monitoring, and multi-robot collaborative operations.

In conclusion, the functional reconstruction of this platform not only expands the application boundaries of biomimetic mechanisms at the engineering implementation level but also provides a competitive technological pathway for meeting the diverse demands of complex marine and scientific missions.

## 6. Conclusions

In practical applications, traditional underwater robots encounter obstacles such as elevated noise levels and inadequate environmental adaptability. In order to enhance the capacity of bionic underwater machines to execute tasks in intricate and unstructured environments, it is imperative to take inspiration from the locomotion mechanisms of aquatic organisms. Consequently, this paper introduces a novel configuration of the bionic underwater robot that is powered by a composite motor and employs the knifefish as a biomimetic template. The robot is endowed with a bionic undulating fin, a single-degree-of-freedom flapping wing, and functions that include efficient propulsion and fine-tuning of posture. The performance evaluation of the robot’s multi-modal underwater movement was conducted using fluid dynamics simulation technology, which established a theoretical foundation for the development of subsequent prototypes and provided feasibility validation. The robot’s feasibility in the multi-modal composite drive mode was further validated by prototype experiments.

The main conclusions are as follows:

(1) A bionic underwater robot platform with an undulating and fluttering multi-modal composite drive was developed and assembled in accordance with the biological locomotion mechanism of the knifefish. The platform is endowed with a bionic undulating fin at the bottom to facilitate efficient propulsion, as well as two pairs of bionic flapping wings on both sides to facilitate posture fine-tuning and auxiliary propulsion, thereby enabling simultaneous composite propulsion.
(2) Kinematic and dynamic models were created for the undulating-flapping composite-driven bionic underwater robot, and motion control and power distribution strategies were suggested. The robot’s characteristics were analyzed during linear and rotational movements using the model. Simulation results indicate that the robot is capable of producing vector propulsion through the utilization of an undulating fin. Furthermore, the higher the flapping frequency, the smaller the turning radius, and the larger the flapping amplitude, the greater the turning radius.
(3) The prototype experiments confirmed the feasibility of the robot’s composite drive mode. By using different motion control strategies, the robot can switch between motion modes, such as forward motion, turning, and buoyancy-sinking, according to the application scenario. In the undulating fin propulsion mode, with a frequency of 2.5 Hz, the maximum speed can reach 0.35 m/s. The flapping wings allow for both linear and turning motions and feature posture adjustment capabilities.

The robot is capable of swiftly switching locomotion modes to accommodate a variety of underwater application scenarios and demonstrates high mobility and strong environmental adaptability. It has a wide range of potential applications in areas such as subsea resource exploration, underwater rescue, and military reconnaissance. Future research will concentrate on two primary areas: first, an in-depth examination of collaborative optimization strategies for multi-modal propulsion modes, which will be accompanied by additional experimental studies to clarify their dynamic coupling effects; and second, the improvement of its degrees of freedom through the use of flexible flapping wings.

## Figures and Tables

**Table 1:** CFD simulation parameters.

Parameter Type	Parameters/Unit	Value
Robot body	Body length, *L*/m	0.74
	Body width, *W*/m	0.14
	Height, *H*/m	0.12
Undulating fin	Fin length, *L_u_*/m	0.93
	Fin width, *W_h_*/m	0.20
	Fin thickness, *t*/m	0
	Wave amplitude, *θ_m_*/°	19.8
Flapping wing	Fin length, *L_f_*/m	0.15
	Fin width, *W_f_* /m	0.08
	Fin thickness, t/m	0
Control parameters	Undulating fin frequency, *f_u_*/Hz	0–2.5
	Flapping wing frequency, *f_p_*/Hz	0–5
	Flapping wing amplitude, *γ*_0_/°	0–60
Dynamic parameters	Weight, *m*/kg	12
	Rotational inertia, *J_xx_*/kg·m^2^	0.08
	Rotational inertia, *J_yy_*/kg·m^2^	0.56
	Rotational inertia, *J_zz_*/kg·m^2^	0.62

**Table 2:** Key structural parameters of the multi-modal composite-driven robot.

Parameter Type	Value
Body dimensions (kg)	810(L) × 135(W) × 420(H)
Overall mass (kg)	12.2
Undulating fin arm length (mm)	140
Undulating fin area (mm^2^)	1.209 × 10^5^
Undulating fin thickness (mm)	2
Flapping wing dimensions (mm)	150(L) × 80(W)

## Data Availability

The data presented in this study are available on request from the corresponding author upon reasonable request.

## Supplementary Materials

The following supporting information can be downloaded at: https://www.mdpi.com/article/10.3390/biomimetics10100678/s1; Video S1: experiment_video_720P.mp4; Video S2: simulation_animation.mp4.
